# Toward a self-driving ultrafast fiber laser

**DOI:** 10.1038/s41377-020-0270-7

**Published:** 2020-02-27

**Authors:** Fanchao Meng, John M. Dudley

**Affiliations:** Université Bourgogne Franche-Comté, Institut FEMTO-ST UMR 6174, Besançon, France

**Keywords:** Fibre lasers, Ultrafast lasers

## Abstract

Femtosecond pulses from an ultrafast mode-locked fiber laser can be optimized in real time by combining single-shot spectral measurements with a smart genetic algorithm to actively control and drive the intracavity dynamics.

The first operation of a laser was recorded by Theodore Maiman in his laboratory notebook on 16 May 1960, and the six decades of subsequent developments in laser science and applications have truly revolutionized society. Only a few years after Maiman’s result, the first indications of the process now known as laser “mode-locking” were reported, and mode-locked lasers producing femtosecond pulses have become essential tools in many important applications. Moreover, ultrafast lasers have been directly associated with several Nobel Prizes: for femtochemistry in 1999, for the broadband frequency comb in 2005, and for chirped pulse amplification in 2018.

Despite these impressive results, however, many emerging applications require ultrafast lasers with precisely tailored temporal and spectral characteristics, and existing approaches to laser design and development have proven to be inadequate. This inadequacy results from the fact that the pulse generation mechanism in ultrafast lasers usually involves complex nonlinear and dispersive propagation effects, and reaching a stable operating regime depends on precisely balancing multiple parameters in a high-dimensional space. This is especially the case for the important class of optical fiber lasers, where, in addition to significant nonlinear and dispersive effects, nonlinear polarization evolution (NPE) is often exploited as the saturable absorption mechanism to drive the mode-locking process. As user demands become more stringent, the alignment of such systems by trial and error is no longer suitable for laser optimization.

Of course, attempts to automate the search for the operating “sweet spot” of NPE ultrafast fiber lasers have been made for a number of years^1–4^. However, the search for a truly self-optimizing laser has recently seen greatly increased progress through the application of advanced algorithmic tools and adaptive feedback and control. This progress has enabled a number of groups to report different approaches to automate optimization of one or more parameters of the laser cavity to reach and maintain a desired operating state^5–13^. A recent publication by Pu et al.^14^ introduced a significant extra novelty by incorporating real-time spectral measurements into the feedback loop. Such measurements have allowed the development of an ultrafast fiber laser system where the spectral characteristics can be automatically tuned, and even noisy regimes of transition instabilities can be accessed in a repeatable way, opening up new possibilities to study fundamental mode-locking dynamics.

The real-time spectral characterization in this work uses the time-stretch dispersive Fourier transform (DFT) technique, which exploits the fact that a temporal signal stretched due to the effect of linear dispersion assumes an intensity profile that is identical to that of its spectrum^15^. Although DFT measurements have become common in characterizing fiber laser instabilities under a wide range of conditions, ref. ^14^ exploited this real-time measurement capability to provide a rapidly updated feedback signal to control the laser operation.

To place this work in context, Fig. 1 shows a generic illustration of how such a smart NPE fiber laser may be configured. Here, in particular, we show the two classes of *control elements* used to date—either the pump power applied to the gain medium or the laser polarization state—as well as the possible output parameters defining the *objective function* used to determine whether the desired stable regime has been reached. To complement this figure, Table 1 summarizes a selection of the state of the art in the field. The optimization strategy used in these previous studies has been generally based on a genetic algorithm, which is also the approach used in ref. ^14^. However, since this recent work uses feedback from a real-time DFT, the algorithm provides direct control over the spectral characteristics of the laser, with impressive programming of pulses with a bandwidth of 10–40 nm and either a Gaussian or triangular spectral shape. The ability of the algorithm to lock on to particular parameter regimes associated with complex transition dynamics is equally notable in view of fundamental studies. Indeed, based on a suitable spectral selection, the authors were able to systematically reach different operating states, such as relaxation oscillation, multisoliton states, and chaos.

**Fig. 1:**
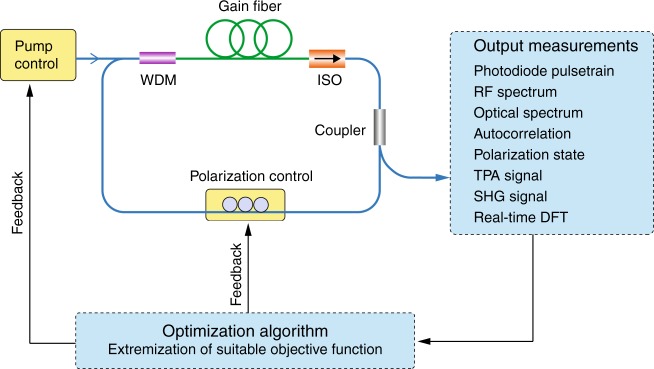
Schematic of a fiber laser cavity with automatic control. WDM wavelength division multiplexer, ISO isolator, RF radiofrequency, TPA two-photon absorption, SHG second harmonic generation, DFT dispersive Fourier transform

**Table 1:** Comparison of automated mode-locking approaches for ultrafast fiber lasers

Laser system	Control element(s)	Objective function(s)	Performance notes
C-Band, anomalous GVD^1^	Polarization control	TPA signal, polarization state	Wavelength and pulse duration selection
C-Band, anomalous and normal GVD^5,6^	Dual polarization control	SHG signal, RF spectral peak	Various pulsed regimes
Yb wave breaking free normal GVD^7^	SLM spectral filtering	Optical spectrum, autocorrelation	CW peak and pedestal suppression
C-band, anomalous GVD^8^	Polarization control, pump power control	Compound: Optical spectrum, peak PD signal, RF spectral peak	Suppression of noise bursts and multipulsing
Yb ANDi, normal GVD^9^	Polarization control	RF spectral peak, optical spectrum	Match to target spectrum, rapid recovery from a perturbation
Yb Fig. 8, normal GVD^12^	Dual independent pump power control	Compound: Autocorrelation, RF spectral peak. Power.	On-demand spectral, temporal, coherence, and energy characteristics
C-Band, anomalous GVD^13^	Polarization control	Temporal pulse counting; FFT pulse train analysis	Pulsed regimes reached via a human-like algorithm
Work by Pu et al. C-Band, anomalous GVD^14^	Polarization control	Real-time spectral measurement using a DFT	Various pulsed regimes, repeatable access to transition dynamics.

*GVD* group velocity dispersion, *TPA* two-photon absorption, *SHG* second harmonic generation, *RF* radio frequency, *SLM* spatial light modulator, *PD* photodiode, *ANDi* all normal dispersion, *FFT* fast Fourier transform, *DFT* dispersive fourier transform

The technical methodology demonstrated in this work using a real-time DFT will very likely be used in many other mode-locked fiber lasers in the future. Another promising area of research will be the expansion of the genetic algorithm approach to use a wider range of tools in the general field of machine learning. Neural networks, for example, have previously been applied to the classification of different regimes of nonlinear propagation in a single-pass geometry^16^, and their extension to active control of mode-locking has already been analyzed theoretically^17^. This extension appears to be a natural next step in the field, and with these techniques, it may even be possible to capture and stabilize particular operating states of mode-locked lasers that currently appear only intermittently in transient regimes of instability^18^.
